# M2-type tumor-associated macrophages promote invasion of canine breast cancer through ADAM9 upregulation

**DOI:** 10.3389/fimmu.2026.1777860

**Published:** 2026-03-11

**Authors:** Fangrong Wu, Chae-Yi Kim, Jeong-Woon Lee, Je-Yoel Cho

**Affiliations:** 1 Department of Biochemistry, Brain Korea 21 Project and Research Institute for Veterinary Science, Seoul National University, College of Veterinary Medicine, Seoul, Republic of Korea; 2 Comparative Medicine Disease Research Center (CDRC), Science Research Center (SRC), Seoul National University, Seoul, Republic of Korea

**Keywords:** ADAM9, breast cancer, cancer stem cells, ECM degration, tumor-associated macrophages

## Abstract

**Introduction:**

Tumor-associated macrophages (TAMs) represent the most abundant immune cell population within the tumor microenvironment and play a critical role in cancer progression. However, the molecular mediators underlying TAM-driven tumor invasion remain incompletely defined. This study investigated whether ADAM9 functions as a key effector of pro-invasive TAM polarization using a canine mammary tumor model integrated with human transcriptomic datasets.

**Methods:**

Transcriptomic analyses were performed using canine and publicly available human datasets. Single-cell RNA sequencing was used to determine cellular localization of ADAM9. IL-4–induced M2 macrophages were evaluated for ADAM9 expression, tumor migration and invasion capacity, extracellular matrix (ECM) degradation, cytoskeletal remodeling, and spheroid destabilization. ADAM9 knockdown and cancer stem cell (CSC)–conditioned medium experiments were conducted to assess mechanistic involvement.

**Results:**

ADAM9 was consistently enriched in M2-polarized TAMs across species and was confirmed to be expressed in TAM populations by single-cell RNA sequencing. IL-4–induced M2 macrophages upregulated ADAM9 and significantly enhanced tumor migration and invasion. ADAM9 knockdown attenuated ECM degradation, reduced MMP9 expression, and disrupted F-actin remodeling. CSC-conditioned medium further induced ADAM9 expression, suggesting its role as a convergent mediator of CSC–macrophage crosstalk. In spheroid models, ADAM9 depletion prevented TAM-mediated spheroid destabilization and suppressed collective invasion.

**Conclusion:**

These findings identify ADAM9 as a key effector of TAM-driven tumor invasion through ECM remodeling and cytoskeletal regulation, and highlight ADAM9 as a promising therapeutic target within the tumor immune microenvironment.

## Introduction

Breast cancer is the most common non-skin malignant tumor among women worldwide and the second leading cause of cancer-related deaths (1). Although the 5-year survival rate for early-stage breast cancer patients can reach 89%, once distant metastasis occurs, the median survival period drops sharply to 1–2 years, and metastatic breast cancer remains a major challenge in clinical treatment (2). Increasing evidence suggests that the tumor microenvironment (TME) plays a significant role in breast cancer metastasis (3, 4).

Within the TME, tumor-associated macrophages (TAMs) are one of the most abundant immune cell types. They can differentiate into M1 (anti-tumor) or M2 (pro-tumor) phenotypes based on microenvironmental signals, with M2 TAMs closely associated with tumor invasion, immune suppression, and angiogenesis (5). Previous studies have shown that there are complex feedback pathways and crosstalk at the gene level between TAMs and tumor cells (6, 7), but the key signaling molecules and their mechanisms of action between the two remain unclear. Macrophage–tumor interactions have been reported to enhance tumor invasion, accompanied by changes in invasion-related genes such as ADAM9 (8).

ADAM proteases (A Disintegrin and Metalloproteinases) are a class of multi-domain transmembrane metalloproteinases that can cleave various cell membrane proteins, thereby activating multiple signaling pathways involved in tumor progression, including the Notch, EGFR, and Eph receptor pathways (9). Among these, ADAM10 and ADAM17 are the most extensively studied family members (10), whereas research on ADAM9 remains relatively limited—especially in the context of immune cell infiltration, immune-checkpoint regulation and tumor microenvironment interactions (11). Previous reports have indicated that ADAM9 mediates cellular responses under stress conditions, regulating processes such as cell adhesion, migration, invasion, proliferation, ECM attachment, and the cleavage of extracellular domains of cell surface proteins (12), and is associated with tumorigenesis and metastasis (13). However, its functional role in TAMs remains unexplored.

Canine mammary tumors (CMT) serve as a naturally occurring breast cancer animal model that shares high similarity with human breast cancer in terms of molecular subtyping (14, 15), hormone receptor expression, and immune microenvironment, and its tumor progression process encompasses the entire spectrum from *in situ* carcinoma to metastatic lesions. Therefore, the canine model provides an important translational platform for studying breast cancer metastasis and TAM-related mechanisms.

In this study, based on public human and dog single-cell data and canine model, we found that ADAM9 is highly expressed in M2 macrophages and promotes breast cancer cell migration and invasion through ECM degradation and structural remodeling. By establishing a co-culture system of macrophages and tumor cells and interfering with ADAM9 expression, we further validated its key role in the tumor-promoting function of TAMs. The findings suggest that ADAM9 is not only a core factor in TAM-mediated migration but may also serve as a potential immunotherapy target.

## Results

### Identification of migration-associated genes in M2 macrophages linked to tumor metastasis

**Figure 1:**
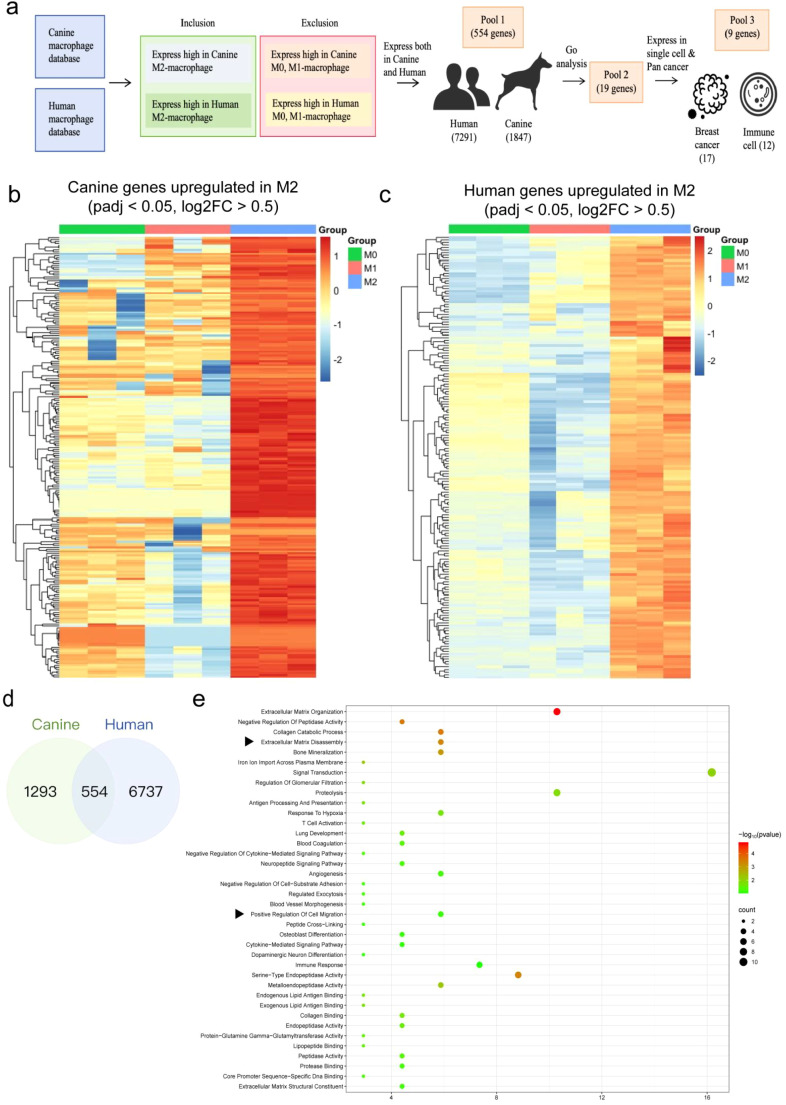
Screening of migration-associated genes based on transcriptomic data from human and canine M2 macrophages. **(a)** Workflow for identifying migration-related differentially expressed genes (DEGs) from publicly available datasets. **(b)** Heatmap illustrating gene expression profiles of human M2 macrophages (n=3), showing markedly elevated expression compared to M0 and M1 phenotypes. Blue to red color gradient denotes low to high expression levels. **(c)** Heatmap displaying the upregulated gene signatures in canine M2 macrophages (n=3). **(d)** Venn diagram indicating the overlap of DEGs between human and canine M2 macrophages. A hypergeometric test was used to assess statistical significance of the intersection of 554 genes (hypergeometric test, p < 0.001). **(e)** GO enrichment of intersecting DEGs performed in DAVID v6.8; migration-related GO terms (GO:0016477) were selected based on FDR < 0.05.

To systematically uncover key genes potentially mediating macrophage-associated migration in breast cancer, we performed a cross-species integrative analysis using macrophage transcriptomic datasets from humans and dogs (**Figure 1a**). Differentially expressed genes (DEGs) were identified by comparing *in vitro*–polarized M2 macrophages with M0 and M1 macrophages, using the criteria |log_2_FC| ≥ 1.5 and adjusted p < 0.05. Human data were derived from a THP-1 monocyte-to-macrophage polarization system, and canine data from a primary monocyte-derived macrophage polarization model. This screening yielded 7,921 and 1,847 upregulated genes in human and canine M2 macrophages, respectively (**Figures 1b, c**). By intersecting these datasets, we identified 554 conserved genes that were consistently upregulated in both species (**Figure 1d**), representing a core transcriptional program associated with M2 polarization. Gene Ontology (GO) enrichment analysis revealed that 19 of these genes (**Supplementary Table 1**) were significantly enriched in migration-related biological processes (**Figure 1e**), suggesting their potential contribution to macrophage-driven metastatic behavior.

To further prioritize candidates with translational relevance, we integrated TCGA pan-cancer and breast cancer single-cell RNA sequencing datasets, ultimately defining 9 migration-associated core genes (ADAM9, ANXA1, SH3KBP1, ZFYVE21, APBB1IP, CTNNA1, PAK2, YWHAG, VCL) that were highly expressed within the tumor microenvironment and correlated with M2 macrophage signatures. Public immune cell transcriptome data showed that ADAM9 was highly expressed in monocytes but barely detected in other immune subsets (**Supplementary Figure 1**) (15). Considering that macrophages can differentiate from circulating monocytes, this pattern supports its lineage-specific enrichment in the monocyte–macrophage axis, consistent with its high expression in M2 macrophages. Among the nine candidates, ADAM9 displayed the most marked M2-specific upregulation in our preliminary qPCR screening and demonstrated the strongest association with invasion-related pathways across multi-omics datasets, providing quantitative and functional support for prioritizing ADAM9. Therefore, ADAM9 was selected for further investigation into tumor–macrophage interactions.

### ADAM9 is highly expressed in M2 macrophages

**Figure 2:**
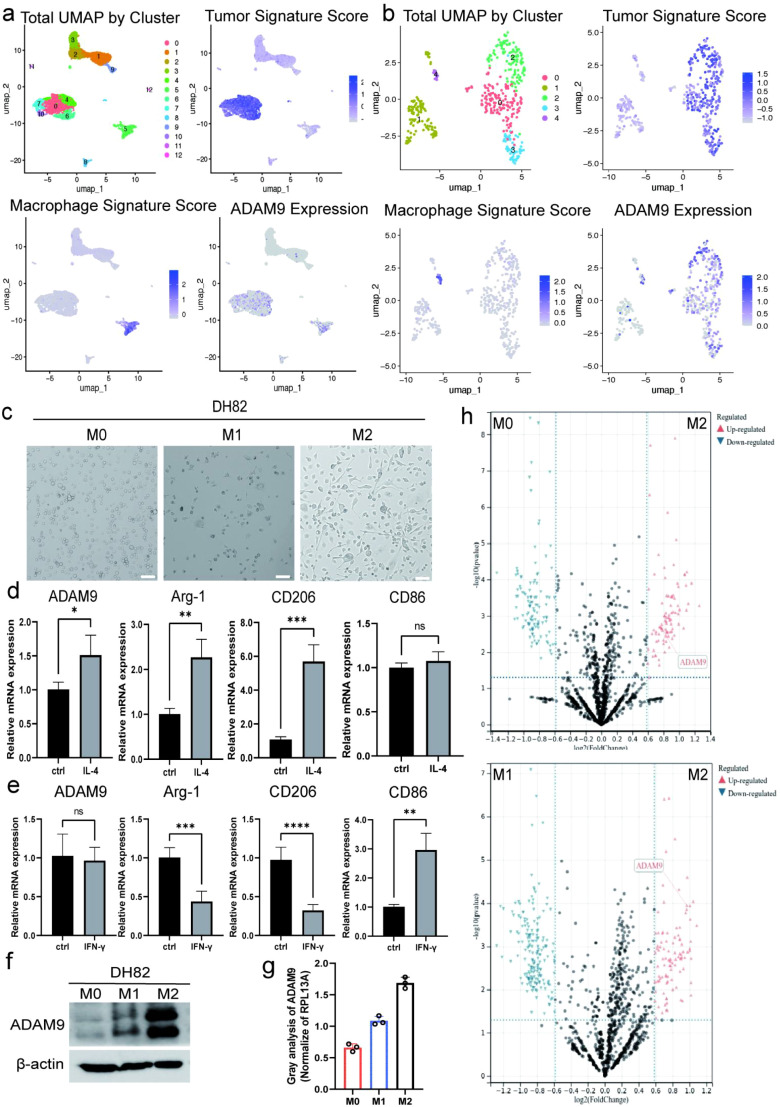
ADAM9 is highly expressed in M2-polarised macrophages. **(a)** tSNE plots from scRNA-seq analysis of primary tumor. **(b)** tSNE plots from scRNA-seq analysis of metastatic specimens. **(c)** Representative bright-field microscopy images of DH82 cells under distinct polarization conditions: unpolarized M0, IFN-γ-induced M1, and IL-4-induced M2. M1 macrophages exhibit amoeboid or round morphology, while M2 macrophages appear fusiform; Scale bar: 100 μm. **(d)** qPCR validation of M1 and M2 marker genes, as well as ADAM9, in DH82 cells following IL-4-induced M2 polarization, using RPL13A as the internal control. **(e)** qPCR analysis of M1, M2 marker genes, and ADAM9 in DH82 cells following IFN-γ-induced M1 polarization, with RPL13A used as the internal control for RNA expression. **(f)** Western blot (WB) analysis of ADAM9 protein levels in M0-, M1-, and M2-polarised macrophages. **(g)** Densitometric quantification of ADAM9 protein levels shown in panel **(f)**, normalized to GAPDH; n = 3 biologically independent samples. **(h)** Volcano plots of DEGs comparing M0 vs. M2 (top) and M1 vs. M2 (bottom); ADAM9 is highlighted among significantly upregulated genes in M2 macrophages (red). All experiments were performed in triplicate unless otherwise specified. Data are presented as means ± SD and were analyzed using two-sided Student’s t test; *P < 0.05; **P < 0.01; ***P < 0.001; ****P < 0.0001; ns, not significant.

To further identify functionally relevant candidate genes, we conducted cell clustering analysis using publicly available single-cell transcriptomic data (GSE264205). Given previous findings indicating reciprocal interactions between tumor cells and TAMs, we analyzed primary tumor samples from patients and observed that ADAM9 was highly expressed in clusters co-expressing tumor and macrophage marker genes (**Figure 2a**). Notably, similar expression patterns were observed in metastatic tumor samples from the same patient, reinforcing the association between ADAM9 and tumor–macrophage interaction (**Figure 2b**). Given the potential role of ADAM9 in intercellular communication, we selected the canine macrophage cell line DH82 as an *in vitro* functional study model. Sequence alignment showed that the homology between canine and human ADAM9 proteins reached 92.4% (**Supplementary Figure 2**), supporting its applicability in tumor mechanism research. Following previous study (16), DH82 cells were stimulated with IL-4 (40 ng/mL) or IFN-γ (20 ng/mL) for 48 hours to induce M2 and M1 polarization, respectively. Brightfield microscopy revealed that IL-4-stimulated cells exhibited typical M2-like spindle-shaped morphology, while the IFN-γ-stimulated group exhibited spherical M1-like morphology (**Figure 2c**), consistent with previous electron microscopy observations (17).

Subsequently, we validated the polarization phenotype via qPCR. In the IL-4-treated group, the M2 marker genes CD206 and Arg-1 were significantly upregulated, while in the IFN-γ group, the M1 marker CD86 expression was enhanced (**Figures 2d, e**). Notably, ADAM9 expression was markedly elevated at both the mRNA and protein levels under IL-4 stimulation (**Figures 2f, g**). Although a modest increase in ADAM9 protein expression was observed in M1 macrophages compared with M0 cells, this change was not paralleled at the transcript level, suggesting potential post-transcriptional regulation. These results show that ADAM9 is consistently and selectively upregulated in M2 macrophages compared with both M1 and M0 states (**Figure 2h**), supporting its potential role as an M2-associated mediator of pro-tumor activity. Given the specific expression of ADAM9 in M2 macrophages, we will next investigate whether it is regulated by tumor cells, particularly cancer stem cell populations, to elucidate the interaction pathways between TAMs and tumor cells.

### CSCs-mediated reprogramming of TAMs leads to enhanced ADAM9 expression

Previous studies have clearly demonstrated that, compared to non-cancer stem cells (NCSCs), cancer stem cells (CSCs) possess a stronger ability to recruit TAMs and remodel the immune microenvironment (18–20). Building on this, we further investigated whether CSCs can specifically regulate ADAM9 expression in TAMs.

**Figure 3:**
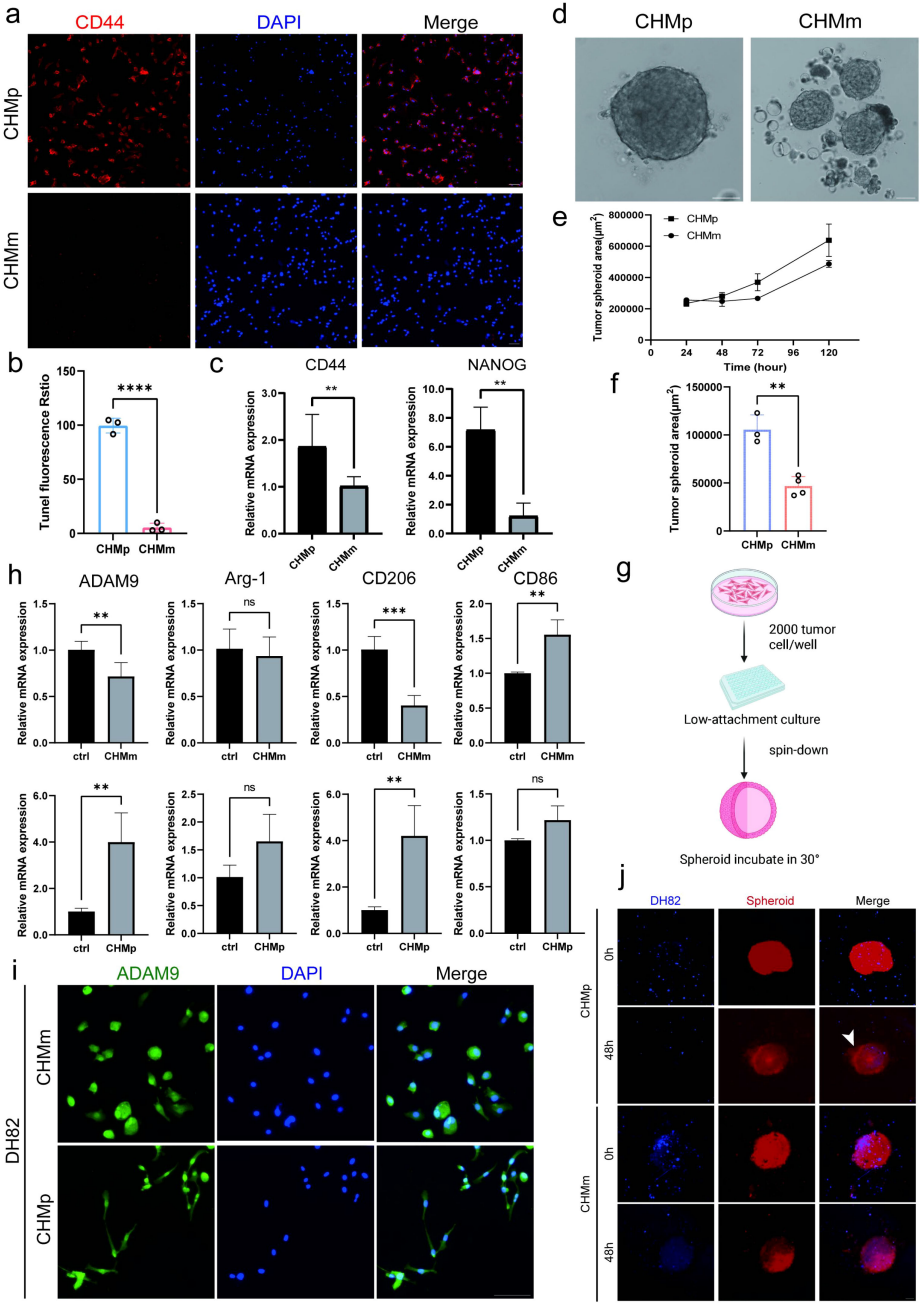
Cancer stem cells upregulate ADAM9 expression and induce M2 polarization of macrophages. **(a)** Immunofluorescence staining of CHMp and CHMm cells for the cancer stem cell marker CD44; Scale bar: 50 μm. **(b)** Quantification of CD44 immunofluorescence intensity in CHMp and CHMm cells. Data are presented as mean ± SEM. **(c)** qPCR analysis of stemness-associated genes in CHMp and CHMm cells and RPL13A was used for internal controls for RNA and protein, respectively. **(d)** Bright-field microscopy images of tumor spheroids formed by CHMp and CHMm cells; Scale bar: 400 μm. **(e)** Quantification of spheroid areas derived from the images shown in **(d)**, comparing CHMp and CHMm spheroids. **(f)** Line graph showing changes in tumor spheroid area over 5 days for CHMp- and CHMm-derived spheroids; n = 3. **(g)** Schematic overview of the spheroid formation and macrophage stimulation workflow. **(h)** qPCR analysis of M1 markers, M2 markers, and ADAM9 in DH82 macrophages following stimulation with conditioned medium (CM) from CHMp (top) or CHMm (bottom) cells. RPL13A was used as the internal control for RNA. ADAM9 induction showed significant upregulation upon CHMp-CM stimulation (p = 0.0047) and CHMm-CM stimulation (p = 0.0045). **(i)** Representative immunofluorescence staining of ADAM9 in DH82 cells co-cultured with tumor cells; Scale bar: 100 μm. **(j)** CellTracker-labelled co-culture system showing tumor spheroids (red) and DH82 macrophages (blue); Scale bar: 200 μm. All experiments were performed in triplicate unless otherwise specified. Data are presented as means ± SD and were analysed using two-sided Student’s t test; *P < 0.05; **P < 0.01; ***P < 0.001; ****P < 0.0001; ns, not significant.

To further explore the impact of CSCs on macrophage function, we selected two canine breast cancer cell lines with high (CHMp) and low (CHMm) stemness phenotypes as *in vitro* models (21). Through CD44 immunofluorescence staining and qPCR analysis, we validated the enrichment of CSC markers in CHMp cells. The results showed that CD44 signaling was significantly enhanced in CHMp cells, indicating a stronger stem cell phenotype (**Figures 3a–c**). Subsequently, we used a three-dimensional spheroid formation assay to functionally validate the differences in stem cell properties between the two cell lines. The results showed that CHMp cells could form dense and structurally intact spheroids, while CHMm cells had weaker spheroid-forming ability (**Figures 3d–f**) (**Supplementary Figure 3**), further confirming that CHMp cells possess stronger stemness characteristics.

Based on this spheroid-based CSC model (**Figure 3g**), conditioned medium (CM) from CHMp or CHMm cells was collected and used to stimulate the dog-derived macrophage cell line DH82. qPCR results showed that CHMp-CM significantly induced DH82 polarization toward the M2 type, accompanied by upregulation of ADAM9 expression; In contrast, CHMm-CM induced upregulation of M1 markers (such as CD86) and did not significantly activate ADAM9 (**Figure 3h**). These results suggest that highly stem cell-like cells may promote the conversion of TAMs to an immunosuppressive phenotype and induce ADAM9 expression by secreting soluble factors. There were obvious morphological differences between the two groups of cells: DH82 cells in the CHMp-CM group exhibited spindle-shaped, elongated morphology, typical of M2-like characteristics, whereas cells in the CHMm-CM group were predominantly round with shortened pseudopodia, exhibiting M1-like morphology (**Figure 3i**) (**Supplementary Figure 4**). There were no significant differences in ADAM9 expression. Spheroids formed by CHMm and CHMp were co-cultured with DH82 macrophages and observed after CellTracker staining. The CHMp spheroid-DH82 co-culture induced a typical “starburst” phenomenon: the spheroid edges disintegrated, accompanied by a large number of migrating cells (22, 23). In the CHMm group, the tumor spheroid structure remained intact, with significantly fewer migrating cells (**Figure 3j**).

In summary, CSCs not only are associated with increased ADAM9 expression in TAMs through secreted factors but may also remodel their polarization characteristics, and this regulation is closely related to the stemness level of cancer cells, providing potential targets for subsequent mechanism studies and therapeutic interventions.

### ADAM9 promotes TAMs-induced tumor cell migration

**Figure 4:**
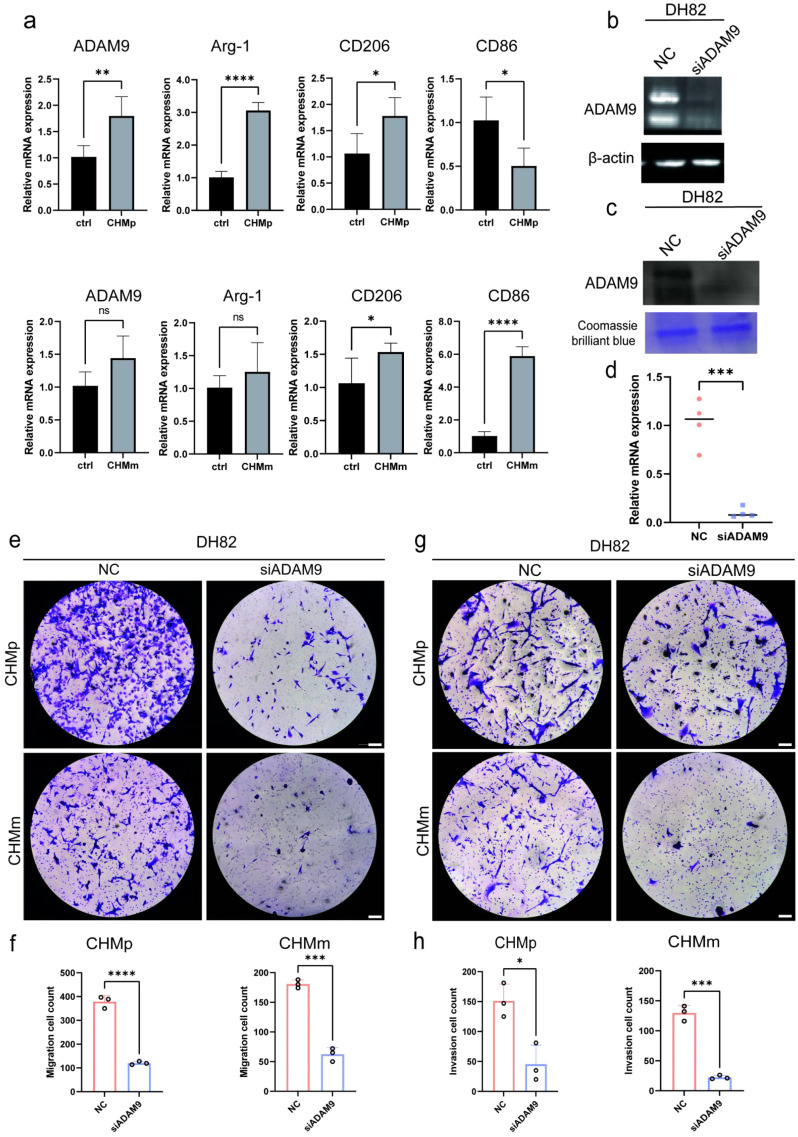
Knockdown of ADAM9 impairs macrophage-mediated cancer cell migration and invasion. **(a)** qPCR analysis of gene expression changes in DH82 macrophages co-cultured with CHMp or CHMm tumor cells and RPL13A was used for internal controls for RNA and protein, respectively. **(b)** WB analysis of ADAM9 protein levels in DH82 macrophages transfected with siADAM9 or control siRNA, with densitometry showing 73% reduction in ADAM9 expression following siADAM9 transfection. **(c)** WB analysis showing reduced ADAM9 secretion following siADAM9 transfection in DH82 macrophages. **(d)** qPCR validation of ADAM9 mRNA expression in macrophages post-siRNA transfection, using RPL13A as the internal control. **(e)** Transwell migration assay performed without Matrigel coating to evaluate the migratory capacity of CHMp and CHMm cancer cells. **(f)** Quantification of invaded cancer cells in the invasion assay shown in **(f)**; n = 3 biologically independent samples. **(g)** Transwell invasion assay using Matrigel-precoated inserts to assess cancer cell invasiveness. **(h)** Quantification of migrated cancer cells in the migration assay shown in **(e)**; n = 3 biologically independent samples. All experiments were performed in triplicate unless otherwise specified. Data are presented as means ± SD and were analyzed using two-sided Student’s t test; *P < 0.05; **P < 0.01; ***P < 0.001; ****P < 0.0001; ns, not significant.

To assess the effect of cancer cells on macrophage polarization status, we used a Transwell co-culture system. In this system, synchronous upregulation of M1 and M2 markers and overall increased ADAM9 expression levels were observed in DH82 cells regardless of co-culture with CHMp or CHMm (**Figure 4a**). This pattern likely reflects a broad activation state in DH82 macrophages under co-culture conditions, in which multiple signaling cues drive concurrent induction of both M1 and M2 markers (24).

ADAM9 was effectively knocked down in DH82 cells via siRNA-mediated transfection. qPCR and Western blot analyses confirmed significant reductions at both the transcriptional and protein levels, including the secreted form of ADAM9 (**Figures 4b–d**). A Transwell co-culture system was then established, with cancer cells seeded in the upper chamber and the lower chamber containing either control DH82 (siCTRL) or ADAM9-knockdown DH82 (siADAM9). Migration assays showed that the number of tumor cells migrating across the membrane was significantly reduced in the siADAM9 group compared to the Ctrl group (**Figures 4e, g**), with an inhibition rate of 58.3% ± 6.2%, suggesting that ADAM9 mediates macrophage-mediated tumor cell migration and invasion.

Furthermore, a Transwell upper chamber coated with matrigel was used to simulate the extracellular matrix barrier *in vivo* to assess the functional role of ADAM9 in tumor invasion. The results showed that ADAM9 knockdown significantly inhibited the invasion ability of both CHMm and CHMp tumor cells, with the inhibition rate of highly stem-like CHMp cells (72.4% ± 5.8%) being significantly higher than that of CHMm cells (41.6% ± 4.3%) (**Figures 4f, h**), suggesting that ADAM9 may enhance tumor cell invasiveness through CSC-related mechanisms. In summary, ADAM9 is not only a key executor molecule for M2-mediated migration/invasion promotion, but its function is also closely related to cancer cell stemness, providing a potential intervention target for targeting the tumor immune microenvironment.

### M2 TAMs promote tumor cell invasion through ADAM9-mediated ECM degradation

**Figure 5:**
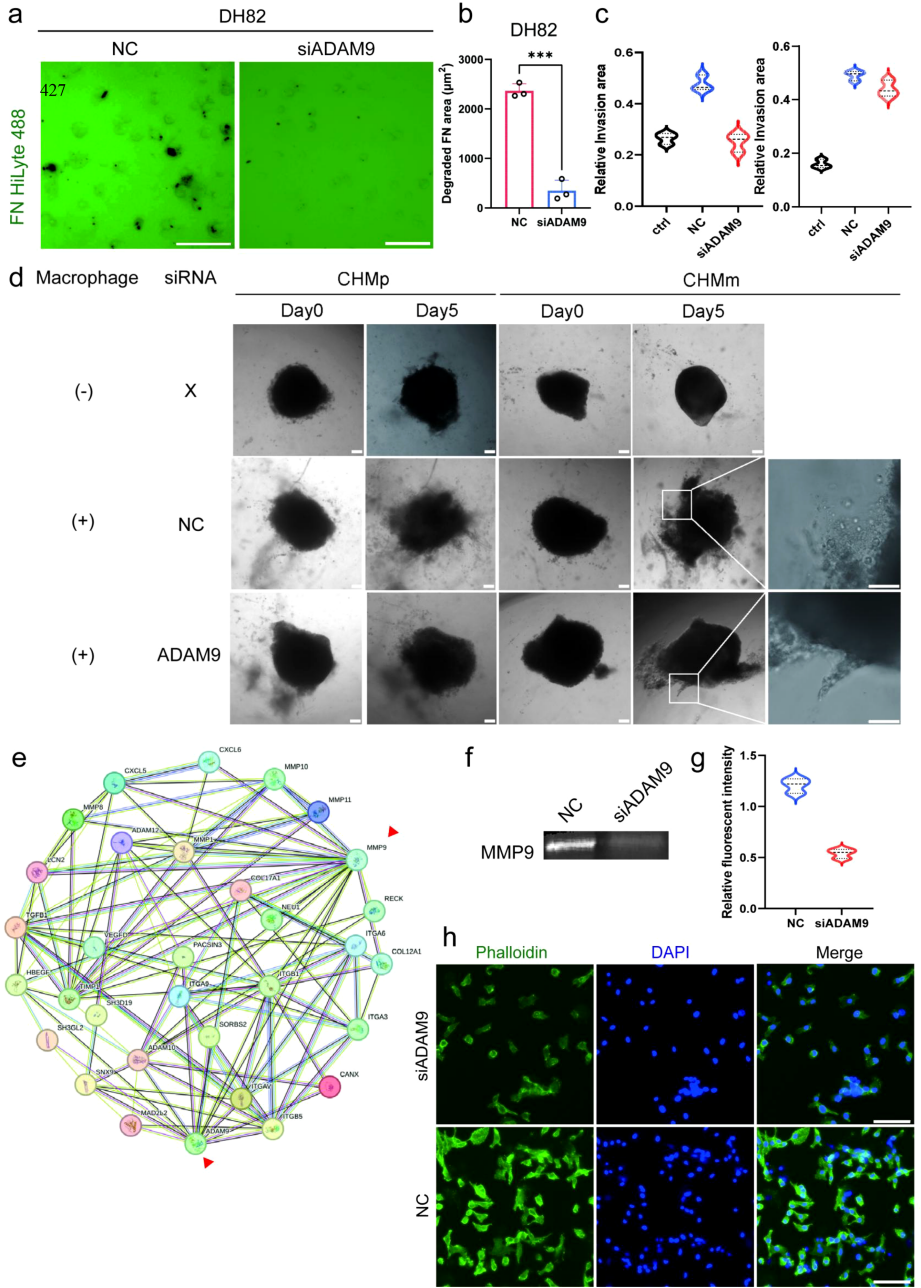
ADAM9 regulates macrophage-driven ECM degradation and tumor invasion. **(a)** Fluorescence images showing ECM degradation capacity of DH82 macrophages transfected with control siRNA (NC) or siADAM9; Scale bar: 100 μm. **(b)** Quantification of fibronectin (FN) degradation area in NC and siADAM9 DH82 macrophages; n = 3. **(c)** Quantification of spheroid invasion areas following 5-day co-culture in CHMp and CHMm models. **(d)** Schematic illustration of tumor spheroid migration under three co-culture conditions. CHMp and CHMm spheroids were cultured either without macrophages, with control siRNA–transfected macrophages (NC), or with siADAM9-transfected macrophages. Images were acquired on day 0 and day 5. **(e)** Protein–protein interaction (PPI) network based on the STRING database showing a predicted association between ADAM9 and MMP9, implicating a potential synergy in ECM remodelling. **(f)** Gelatin zymography analysis of MMP in the supernatants collected from DH82 macrophages. **(g)** Quantification of F-actin intensity in NC and siADAM9 DH82 macrophages stained with phalloidin; n=3. **(h)** Immunofluorescence staining of F-actin (phalloidin) in DH82 cells, showing reduced cytoskeletal signal following siADAM9 treatment; Scale bar: 20 μm. All experiments were performed with at least three biologically independent replicates unless otherwise specified. Data are presented as means ± SD and were analyzed using two-sided Student’s t test; ***P < 0.001.

To further clarify the role of ADAM9 in TAM-mediated tumor-promoting functions—particularly its involvement in extracellular matrix (ECM) remodeling—we first examined matrix-degrading activity using a FITC–gelatin assay. Mammary tumor cell lines (CHMp and CHMm) alone showed minimal ECM degradation (**Supplementary Figure 5**), whereas IL-4–induced M2-type DH82 macrophages efficiently digested the fluorescent matrix. Importantly, silencing ADAM9 markedly impaired this degradative capacity, as reflected by a significant decrease in the area of gelatin breakdown (**Figures 5a, b**). Together, these results indicate that ADAM9 is required for the full ECM-degrading capacity of M2-polarized TAMs.

To recapitulate the invasive dynamics within the tumor microenvironment, we employed a spheroid–macrophage co-culture model. In the absence of macrophages, CHMp spheroids displayed outward dispersal, while CHMm spheroids remained compact (**Figure 5d**). Upon co-culture with control DH82 macrophages, CHMm spheroids acquired invasive characteristics, confirming that macrophages promote tumor invasion. Notably, when co-cultured with ADAM9-silenced macrophages, invasion persisted but shifted from single-cell migration via pseudopodia to a more cohesive, collective protrusive pattern (**Figure 5c**), indicating that ADAM9 contributes to both invasion extent and invasion mode. Such mode-switching is consistent with the known plasticity of cancer invasion, which is shaped by ECM architecture and proteolytic activity (25, 26).

At the molecular level, MMP-9 is a principal enzyme responsible for ECM degradation. Previous studies have linked elevated ADAM9 expression to enhanced MMP-9 secretion. Protein–protein interaction analysis via STRING identified a potential connection between ADAM9 and MMP-9 (**Figure 5e**). Consistently, gelatin zymography showed a reduction in overall MMP-9–associated gelatinolytic activity in supernatants from siADAM9-treated DH82 cells (**Figure 5f**). Because pro- and active-MMP-9 bands were not clearly separated, the decrease reflects total gelatinolytic activity rather than specific regulation of MMP-9 activation. These results support the notion that ADAM9 contributes to protease-dependent matrix remodeling.

Beyond matrix degradation, actin cytoskeleton integrity is essential for cell motility and morphological adaptation. F-actin visualized by phalloidin staining was markedly diminished in ADAM9-knockdown macrophages, indicating altered cytoskeletal organization (**Figures 5g, h**). This reduction may reflect changes in cell adhesion or motile behavior rather than a direct effect on actin polymerization. This aligns with prior findings that ADAM family proteins and MMP activity coordinately influence focal adhesion dynamics and cytoskeletal remodeling during migration.

In summary, our data suggest that ADAM9 exerts a multilayered regulatory role in M2-driven tumor invasion: by enhancing ECM degradation, influencing the mode of invasion (shifting between collective and individual), and maintaining actin cytoskeletal structure. ADAM9 thus acts as a critical node bridging immune microenvironment, ECM remodeling, and invasion strategy, with promising therapeutic implications.

## Discussion

Metastasis remains the primary cause of mortality in breast cancer, and the presence of invasive tumor phenotypes driven by TAMs represents a major therapeutic challenge (27). Although clinical data consistently demonstrate that dense infiltration of M2 TAMs correlates with poor prognosis, the precise mechanism by which CSCs communicate with macrophages to reprogram the TME has remained unclear (28). In this study, we identified a novel immune-escape and metastasis-promoting mechanism, which can be a potential ‘CSCs-TAM-ADAM9’ interaction model.” Our findings suggest a possible model by which CSCs influence macrophages: by secreting factors that upregulate ADAM9 in M2 TAMs, CSCs create a pro-metastatic niche that facilitates ECM degradation, cytoskeletal remodeling, and invasion plasticity.

Mechanistically, ADAM9 emerges as more than a “sheddase” (29). Previous studies have reported that ADAM9 can cooperate with MMP-9 to promote ECM degradation in invasive cancer (30). Consistently, we observed a predicted ADAM9–MMP-9 association in PPI analysis and reduced MMP-9 activity after ADAM9 silencing, suggesting that ADAM9 may support MMP-9–dependent matrix remodeling. Our data suggest that ADAM9 is associated with enhanced MMP-9-mediated ECM-degrading activity and contributes to the stabilization of F-actin architecture in tumor cells, thereby promoting both invasion efficiency and migration persistence, consistent with previous reports (31, 32). However, the reduction in F-actin observed upon ADAM9 knockdown may not exclusively reflect a direct effect on actin polymerization; alterations in cell adhesion or motile behavior may also contribute. Strikingly, ADAM9 knockdown disrupted pseudopod formation, impaired macrophage-mediated gelatin degradation, and markedly reduced tumor cell invasiveness. These findings place ADAM9 as a pivotal executor of TAM pro-tumor function, expanding the biological significance of ADAM proteins from tumor cell–intrinsic enzymes to immune cell–derived regulators of metastatic progression.

Notably, we found that CSCs regulate both the magnitude and the mode of invasion through ADAM9. Highly stem-like CHMp cells induced robust ADAM9 expression in TAMs through soluble cues, whereas less stem-like CHMm cells required direct contact to trigger a similar response. Using a three-dimensional co-culture model, we observed that TAMs endowed non-invasive spheroids with invasive properties and reshaped their migration pattern—from individual cell dissemination to collective invasion characterized by cohesive, front-led protrusions. This transition indicates that ADAM9 can act as a molecular switch controlling invasion modality, integrating cytoskeletal regulation with intercellular coordination. Such plasticity may enable tumors to alternate between single-cell and collective modes depending on microenvironmental constraints, thereby enhancing metastatic adaptability.

Despite these advances, several questions remain unresolved. The upstream signals governing ADAM9 transcription in TAMs have yet to be fully elucidated, and how ADAM9 integrates with canonical ECM-remodeling pathways—such as MMP cascades or the integrin–FAK axis—requires deeper exploration (33, 34). Moreover, while our data establish a strong preclinical foundation, validation in orthotropic and metastatic models is essential to clarify ADAM9’s role *in vivo* and to evaluate therapeutic feasibility.

From a translational perspective, our findings suggest that disrupting the CSCs-ADAM9-TAM axis could provide a novel avenue for anti-metastatic therapy. Targeting ADAM9 directly, or reprogramming TAMs to a non-permissive phenotype, may blunt tumor invasion at its root. Future strategies combining ADAM9 inhibitors with immune checkpoint blockade or TAM-modulating agents hold promise for reshaping the TME and restraining metastatic dissemination. However, because this study relied on a single macrophage model (DH82), it may not fully capture the heterogeneity of M2-like TAM subsets (M2a, M2b, M2c, M2d) present in the tumor microenvironment, highlighting an important consideration for translational application.

In summary, this work establishes ADAM9 as a key effector in M2 macrophages that orchestrates CSC-driven invasion and invasion-pattern switching. By defining the CSCs-ADAM9-TAM axis, we provide new mechanistic insight into tumor immune microenvironment regulation and propose a potential therapeutic target to address metastasis, the most lethal hallmark of breast cancer.

## Materials and methods

### Public data and bioinformatic analysis

The GEO dataset GSE213204 (16) was retrieved from the GEO database (http://www.ncbi.nlm.nih.gov/geo), and the corresponding RNA expression data were downloaded from PLOS ONE dataset (35) for comparative analysis of RNA levels among M0, M1, and M2 macrophages. Gene Ontology (GO) functional annotation and Kyoto Encyclopedia of Genes and Genomes (KEGG) pathway enrichment analyses were performed using the web-based DAVID v6.8 platform.

### Single-cell analysis

Raw gene expression matrices from GSE264205 were analyzed using Seurat (v3.0.0) in R (v3.5.2). Cells with >25% mitochondrial RNA content were excluded. Remaining cells were normalized using the SCTransform method. Principal component analysis (PCA) and UMAP were used for dimensionality reduction. Datasets were integrated using FindIntegrationAnchors and IntegrateData functions. Clustering was performed at a resolution of 0.1. Marker genes were identified using FindAllMarkers.

### Cell culture and macrophage polarization

Canine macrophage cell line DH82 (ATCC CRL-10389) and canine mammary adenocarcinoma cell lines CHMp and CHMm (21) were cultured in high-glucose DMEM (Hyclone, SH30243.01) supplemented with 10% FBS (Sigma, F2442)and 1% penicillin-streptomycin (Sigma–Aldrich, G1272) at 37 °C with 5% CO2. Cells were confirmed mycoplasma-free. For polarization, DH82 were treated with 20 ng/mL IFN-γ for M1, or 40 ng/mL IL-4 for M2 polarization. M0 controls were cultured without stimulation. On day 7, cells were harvested for downstream assays.

### RNA isolation and qRT-PCR

RNA isolation and real-time quantitative RT–PCR were performed as previously described (35). Total RNA was extracted using TRIzol (Ambion, 15596018) and total RNA was extracted through sequential phase separation with chloroform and precipitation with isopropanol. The RNA pellet was washed with 75% ethanol, air-dried, and dissolved in DEPC water (Biosesang, WR2004-050-00). For reverse transcription, 1μg of total RNA was used as a template and converted into complementary DNA (cDNA) using the cDNA Strand Synthesis Master Mix (Cellsafe, CDS-400) following the manufacturer’s instructions. Gene expression was quantified using SYBR Green (Invitrogen, S33102) and GoTaq (Promega, M8298). All the specific primers used for PCR amplification are listed in **Supplementary Table 2**. Gene expression was normalized to RPL13A, which shows stable expression during macrophage polarization.

### Western blotting

Protein extraction and Western blotting were carried out as described previously (35). For secreted protein detection, culture supernatants were treated with protease inhibitors. Protein lysates were separated by SDS-PAGE and transferred to PVDF membranes. Molecular weight separation was assessed using a prestained protein ladder (Thermo, 26616). Membranes were blocked with 5% skim milk (Millipore, 115363) in TBS-T for 1 h at room temperature, followed by incubation with primary antibodies against ADAM9 (1:3000, GeneTex, GTX130081) and β-actin (1:3000, Santa Cruz, sc-47778). Goat anti-rabbit (1:3000, Bethyl, A120-101P) and goat anti-mouse (1:3000, Bethyl, A90-116P) antibodies were used as secondary antibodies.

### Immunofluorescence staining

Cells were fixed with 4% paraformaldehyde (Biosesang, P2031), permeabilized with 0.02% Triton X-100 (Sigma, X100) and blocked with 5% BSA (Milteny Biotec, 130-091-376). Primary antibodies ADAM9 (1:300), CD44, Phalloidin (1:300, Sigma, 65906) and DAPI (1:300, Enzynomics, EBD001) were applied. Goat Anti-Rabbit Alexa Fluor 488 (1:300, Abcam, ab150077) and Goat Anti-Mouse Alexa Fluor 594 (1:300, Abcam, ab150116), were used as secondary antibodies. F-actin was stained with phalloidin. Imaging was performed on an Olympus spinning disk confocal microscope. Fluorescence intensity was quantified using ImageJ. Mean fluorescence intensity was measured from ROIs corresponding to individual cells after background subtraction. 20 cells were analyzed per condition across three biological replicates, and values were normalized to the control group.

### Transwell migration and invasion assay

For migration assays, 2.5 x 10^5^ cells in serum-free medium were seeded in the upper chamber of a Transwell insert with an 8-μm pore size (Corning, 3422). For invasion assays, chambers were pre-coated with Matrigel. After 24–48 h, membranes were fixed with 4% paraformaldehyde solution (Biosesang, P2031) for 15 min at room temperature and stained with 0.2% crystal violet (Sigma, C0775). Cells on the lower surface were counted under a microscope.

### siRNA transfection

Negative control siRNA (Bioneer, SDH-1001) and canine ADAM9–targeting siRNA designed and synthesized by Bioneer were utilized for knockdown experiments. The sequences were: 5’-CUGCUUUUGUCUUCAUCAA-3’ and 5’-UUGAUGAAGACAAAAGCAG-3’. Alternatively, siRNA transfection was performed using the NEPA21 Super Electroporator (Nepa Gene) via cuvette electroporation, according to the manufacturer’s instructions. The specific electroporation conditions for the poring pulse and transfer pulse are listed in **Supplementary Table 3**.Transfection efficiency was assessed after 48–72 h via western blot and degradation assays.

### Conditioned medium

For conditioned medium (CM) experiments, supernatants from CHMp or CHMm cells cultured for 48 h were collected and centrifuged at 1300 rpm to remove cellular debris, followed by filtration through a 0.22-μm membrane. The resulting CM was mixed 1:1 with complete DMEM (final CM concentration: 50%) and applied to DH82 macrophages. The treatment medium was refreshed every 24 h.

### 3D co-culture invasion assay

Spheroids of CHMm or CHMp were formed in ultra-low attachment plates for 5 days and embedded in a mixture of Vitrogel and medium (2:1). DH82 macrophages were added to each spheroid at a macrophage:tumor cell ratio of 5:1 and co-cultured for 48 h.

### Matrix degradation assay

Coverslips were pre-coated by 200 μg/mL fluorescent gelatin (Invitrogen, G13187). Cells were seeded and incubated for 12–24 h. ECM degradation zones were quantified by ImageJ using fluorescence intensity and area from at least seven random fields.

### Statistical analysis

All experiments were repeated independently at least three times. Data were presented as mean ± SD. Statistical significance was determined using Student’s t-test or one-way ANOVA with Tukey’s *post hoc* test in GraphPad Prism 9.3.1. A p-value < 0.05 was considered statistically significant.

## Data Availability

The datasets presented in this study can be found in online repositories. The names of the repository/repositories and accession number(s) can be found in the article/**Supplementary Material**.

## References

[1] Bray F Laversanne M Sung H Ferlay J Siegel RL Soerjomataram I . Global cancer statistics 2022: GLOBOCAN estimates of incidence and mortality worldwide for 36 cancers in 185 countries. CA Cancer J Clin. (2024) 74:229–63. doi: 10.3322/caac.21834, PMID: 38572751

[2] Kim MY . Breast cancer metastasis. Adv Exp Med Biol. (2021) 1187:183–204. doi: 10.1007/978-981-32-9620-6_9, PMID: 33983579

[3] Liu M Ren Y Zhou Z Yang J Shi X Cai Y . The crosstalk between macrophages and cancer cells potentiates pancreatic cancer cachexia. Cancer Cell. (2024) 42:885–903.e4. doi: 10.1016/j.ccell.2024.03.009, PMID: 38608702 PMC11162958

[4] Xu J Gao F Liu W Guan X . Cell-cell communication characteristics in breast cancer metastasis. Cell Commun Signal. (2024) 22:55. doi: 10.1186/s12964-023-01418-4, PMID: 38243240 PMC10799417

[5] Mehta AK Kadel S Townsend MG Oliwa M Guerriero JL . Macrophage biology and mechanisms of immune suppression in breast cancer. Front Immunol. (2021) 12:643771. doi: 10.3389/fimmu.2021.643771, PMID: 33968034 PMC8102870

[6] Zhang Q Tsui YM Zhang VX Lu AJ Lee JM Lee E . Reciprocal interactions between Malignant cells and macrophages enhance cancer stemness and M2 polarization in HBV-associated hepatocellular carcinoma. Theranostics. (2024) 14:892–910. doi: 10.7150/thno.87962, PMID: 38169544 PMC10758064

[7] Li H Yang P Wang J Zhang J Ma Q Jiang Y . HLF regulates ferroptosis, development and chemoresistance of triple-negative breast cancer by activating tumor cell-macrophage crosstalk. J Hematol Oncol. (2022) 15:2. doi: 10.1186/s13045-021-01223-x, PMID: 34991659 PMC8740349

[8] Shen Z Kauttu T Cao J Seppänen H Vainionpää S Ye Y . Macrophage coculture enhanced invasion of gastric cancer cells via TGF-β and BMP pathways. Scand J Gastroenterol. (2013) 48:466–72. doi: 10.3109/00365521.2013.772226, PMID: 23517295

[9] Camodeca C Cuffaro D Nuti E Rossello A . ADAM metalloproteinases as potential drug targets. Curr Med Chem. (2019) 26:2661–89. doi: 10.2174/0929867325666180326164104, PMID: 29589526

[10] Seegar TC Blacklow SC . Domain integration of ADAM family proteins: Emerging themes from structural studies. Exp Biol Med (Maywood). (2019) 244:1510–9. doi: 10.1177/1535370219865901, PMID: 31333048 PMC6920674

[11] AmeliMojarad M AmeliMojarad M Wang J Tavakolpour V Shariati P . A pan-cancer study of ADAM9’s immunological function and prognostic value particularly in liver cancer. Sci Rep. (2024) 14:26862. doi: 10.1038/s41598-024-76049-x, PMID: 39505907 PMC11541887

[12] Chandrasekera P Perfetto M Lu C Zhuo M Bahudhanapati H Li J . Metalloprotease ADAM9 cleaves ephrin-B ligands and differentially regulates Wnt and mTOR signaling downstream of Akt kinase in colorectal cancer cells. J Biol Chem. (2022) 298:102225. doi: 10.1016/j.jbc.2022.102225, PMID: 35780836 PMC9358476

[13] Micocci KC Moritz MN Lino RL Fernandes LR Lima AG Figueiredo CC . ADAM9 silencing inhibits breast tumor cells transmigration through blood and lymphatic endothelial cells. Biochimie. (2016) 128-129:174–82. doi: 10.1016/j.biochi.2016.08.006, PMID: 27554339

[14] Abdelmegeed SM Mohammed S . Canine mammary tumors as a model for human disease. Oncol Lett. (2018) 15:8195–205. doi: 10.3892/ol.2018.8411, PMID: 29928319 PMC6004712

[15] Gray M Meehan J Martínez-Pérez C Kay C Turnbull AK Morrison LR . Naturally-occurring canine mammary tumors as a translational model for human breast cancer. Front Oncol. (2020) 10:617. doi: 10.3389/fonc.2020.00617, PMID: 32411603 PMC7198768

[16] Lyu Q Veldhuizen EJA Ludwig IS Rutten VPMG van Eden W Sijts AJAM . Characterization of polarization states of canine monocyte derived macrophages. PloS One. (2023) 18:e0292757. doi: 10.1371/journal.pone.0292757, PMID: 37939066 PMC10631683

[17] Heinrich F Lehmbecker A Raddatz BB Kegler K Tipold A Stein VM . Morphologic, phenotypic, and transcriptomic characterization of classically and alternatively activated canine blood-derived macrophages in *vitro*. PloS One. (2017) 12:e0183572. doi: 10.1371/journal.pone.0183572, PMID: 28817687 PMC5560737

[18] Allavena P Digifico E Belgiovine C . Macrophages and cancer stem cells: a malevolent alliance. Mol Med. (2021) 27:121. doi: 10.1186/s10020-021-00383-3, PMID: 34583655 PMC8480058

[19] Chu X Tian W Ning J Xiao G Zhou Y Wang Z . Cancer stem cells: advances in knowledge and implications for cancer therapy. Signal Transduct Target Ther. (2024) 9:170. doi: 10.1038/s41392-024-01851-y, PMID: 38965243 PMC11224386

[20] Mierke CT . The versatile roles of ADAM8 in cancer cell migration, mechanics, and extracellular matrix remodeling. Front Cell Dev Biol. (2023) 11:1130823. doi: 10.3389/fcell.2023.1130823, PMID: 36910158 PMC9995898

[21] Murai K Nakagawa T Endo Y Kamida A Yoshida K Mochizuki M . Establishment of a pair of novel cloned tumour cell lines with or without metastatic potential from canine mammary adenocarcinoma. Res Vet Sci. (2012) 93:468–72. doi: 10.1016/j.rvsc.2011.06.012, PMID: 21714977

[22] Nazari SS Doyle AD Yamada KM . Mechanisms of Basement Membrane Micro-Perforation during Cancer Cell Invasion into a 3D Collagen Gel. Gels. (2022) 8(9):567. doi: 10.20944/preprints202208.0242.v1, PMID: 36135279 PMC9498339

[23] Singh K Gautam PK . Macrophage infiltration in 3D cancer spheroids to recapitulate the TME and unveil interactions within cancer cells and macrophages to modulate chemotherapeutic drug efficacy. BMC Cancer. (2023) 23:1201. doi: 10.1186/s12885-023-11674-9, PMID: 38062442 PMC10701966

[24] Kerneur C Cano CE Olive D . Major pathways involved in macrophage polarization in cancer. Front Immunol. (2022) 13:1026954. doi: 10.3389/fimmu.2022.1026954, PMID: 36325334 PMC9618889

[25] Pandya P Orgaz JL Sanz-Moreno V . Modes of invasion during tumour dissemination. Mol Oncol. (2017) 11:5–27. doi: 10.1002/1878-0261.12019, PMID: 28085224 PMC5423224

[26] Wu JS Jiang J Chen BJ Wang K Tang YL Liang XH . Plasticity of cancer cell invasion: Patterns and mechanisms. Transl Oncol. (2021) 14:100899. doi: 10.1016/j.tranon.2020.100899, PMID: 33080522 PMC7573380

[27] Kzhyshkowska J Shen J Larionova I . Targeting of TAMs: can we be more clever than cancer cells? Cell Mol Immunol. (2024) 21:1376–409. doi: 10.1038/s41423-024-01232-z, PMID: 39516356 PMC11607358

[28] Verona F Di Bella S Schirano R Manfredi C Angeloro F Bozzari G . Cancer stem cells and tumor-associated macrophages as mates in tumor progression: mechanisms of crosstalk and advanced bioinformatic tools to dissect their phenotypes and interaction. Front Immunol. (2025) 16:1529847. doi: 10.3389/fimmu.2025.1529847, PMID: 39981232 PMC11839637

[29] Li Y Zhao X Liu Q Liu Y . Bioinformatics reveal macrophages marker genes signature in breast cancer to predict prognosis. Ann Med. (2021) 53:1019–31. doi: 10.1080/07853890.2021.1914343, PMID: 34187256 PMC8253219

[30] Zhao S Wang J Cao Z Gao L Zheng Y Wang J . miR-126a-3p induces proliferation, migration and invasion of trophoblast cells in pre-eclampsia-like rats by inhibiting A Disintegrin and Metalloprotease 9. Biosci Rep. (2019) 40(7):BSR-20191271. doi: 10.1042/BSR20191271, PMID: 31789346 PMC6923329

[31] Zigrino P Steiger J Fox JW Löffek S Schild A Nischt R . Role of ADAM-9 disintegrin-cysteine-rich domains in human keratinocyte migration. J Biol Chem. (2007) 282:30785–93. doi: 10.1074/jbc.M701658200, PMID: 17704059

[32] Micocci KC Martin AC Montenegro Cde F Durante AC Pouliot N Cominetti MR . ADAM9 silencing inhibits breast tumor cell invasion in *vitro*. Biochimie. (2013) 95:1371–8. doi: 10.1016/j.biochi.2013.03.001, PMID: 23499592

[33] Xu B Lefringhouse J Liu Z West D Baldwin LA Ou C . Inhibition of the integrin/FAK signaling axis and c-Myc synergistically disrupts ovarian cancer Malignancy. Oncogenesis. (2017) 6:e295. doi: 10.1038/oncsis.2016.86, PMID: 28134933 PMC5294249

[34] Li S Sampson C Liu C Piao HL Liu HX . Integrin signaling in cancer: bidirectional mechanisms and therapeutic opportunities. Cell Commun Signal. (2023) 21:266. doi: 10.1186/s12964-023-01264-4, PMID: 37770930 PMC10537162

[35] Lee D Kim DW Yoon S Nam AR Lee KH Nam KH . CXCL5 secreted from macrophages during cold exposure mediates white adipose tissue browning. J Lipid Res. (2021) 62:100117. doi: 10.1016/j.jlr.2021.100117, PMID: 34537202 PMC8512628

