# Supplementary material for: M2-type tumor-associated macrophages promote invasion of canine breast cancer through ADAM9 upregulation
Source: Front Immunol. 2026 Mar 11;17:1777860. doi: 10.3389/fimmu.2026.1777860 (PMC13014407; doi:10.3389/fimmu.2026.1777860)
Supplement: Supplementary file 1 [file DataSheet1.docx]

**Supplementary Materials**

**Supplementary Figure 1** ADAM9 expression across immune cell types

**Supplementary Figure 2** Analysis of the homology between dog and human

**Supplementary Figure 2** Growth changes in CHMm and CHMp spheroids over 5 days

**Supplementary Figure 4** Comparison of spheroid invasiveness between CHMm and CHMp

**Supplementary Figure 5** ECM degradation of CHMm and CHMp

**Supplementary Table 1** Gene list obtained through screening

**Supplementary Table 2** The table of specific primers for PCR amplification is listed.

**Supplementary Table 3** The table of specific electroporation conditions for canine DH82 cell lines is listed.

**Supplementary Figure 1** ADAM9 expression across immune cell types


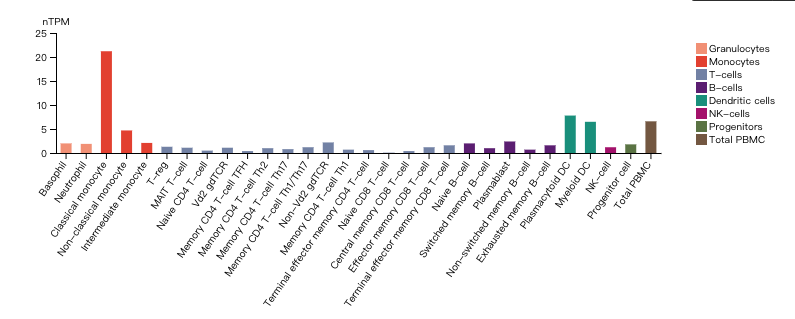


**Supplementary Figure 2** Analysis of the homology between dogs and humans

**
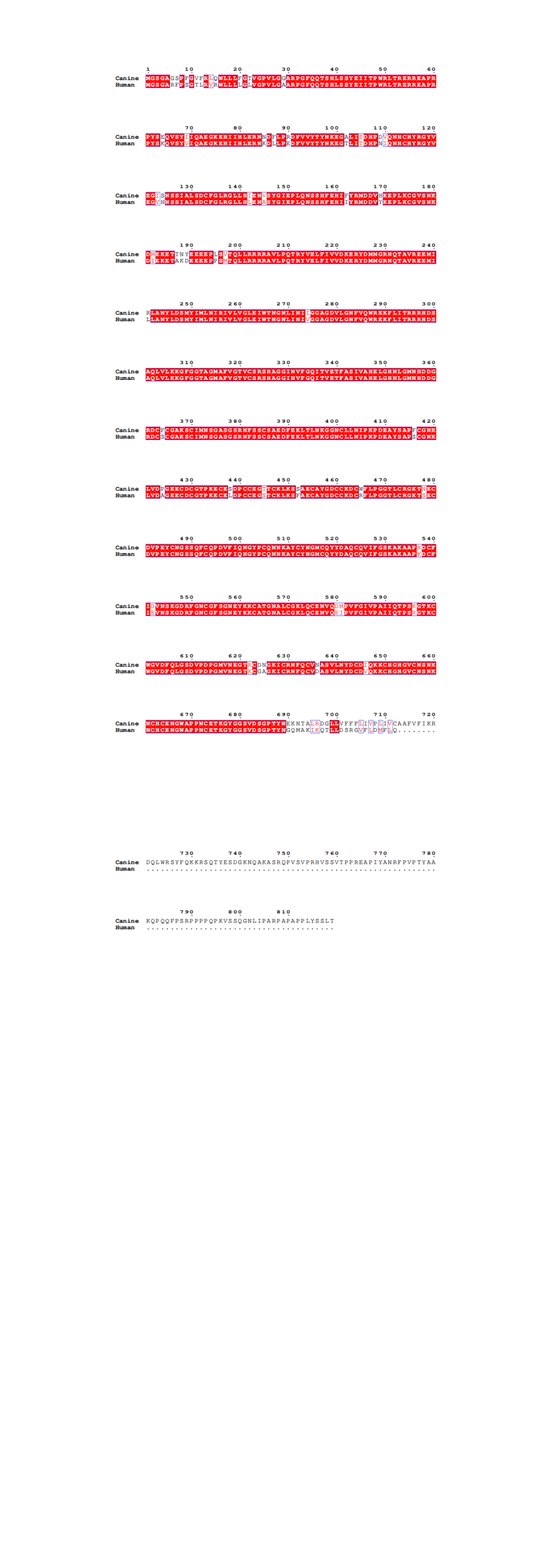
**

**Supplementary Figure 3 Growth changes in CHMm and CHMp spheroids over 5 days**

**
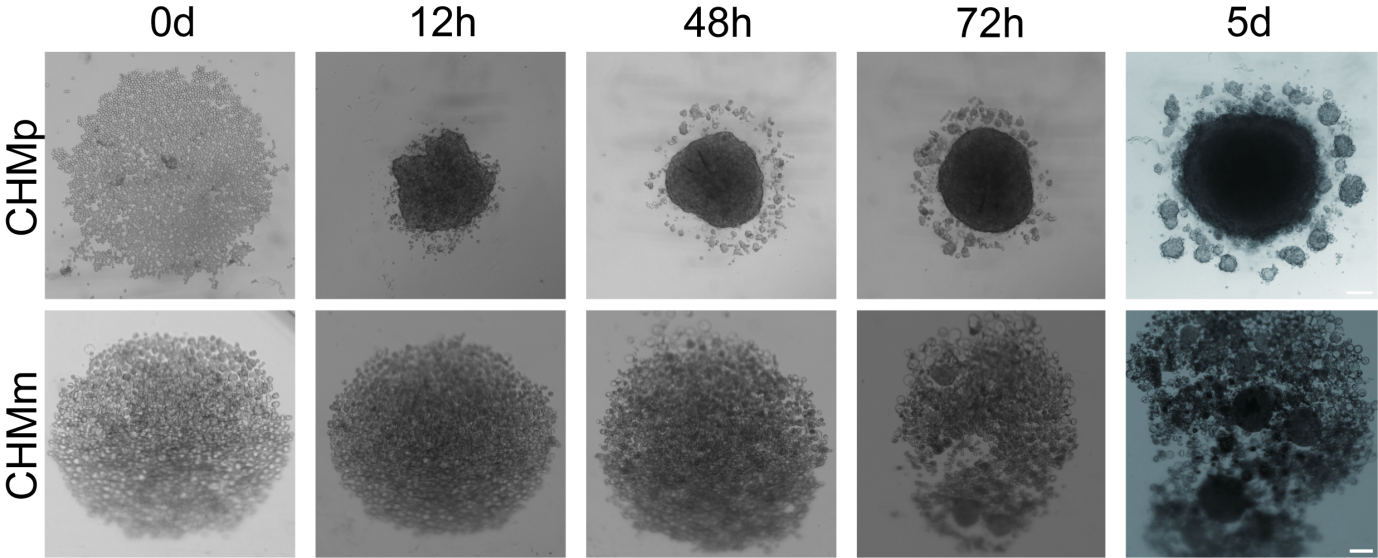
**

**Scale bar: 100 μm**

**Supplementary Figure 4 Comparison of spheroid invasiveness between CHMm and CHMp**

**
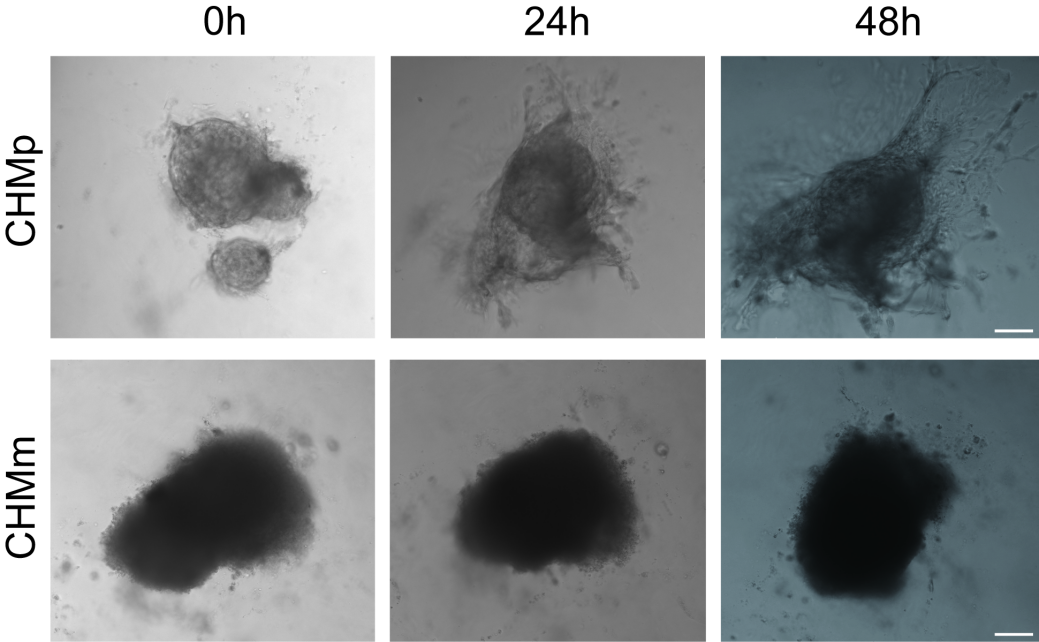
**

**Scale bar: 200 μm**

**Supplementary Figure 5** ECM degradation of CHMm and CHMp

**
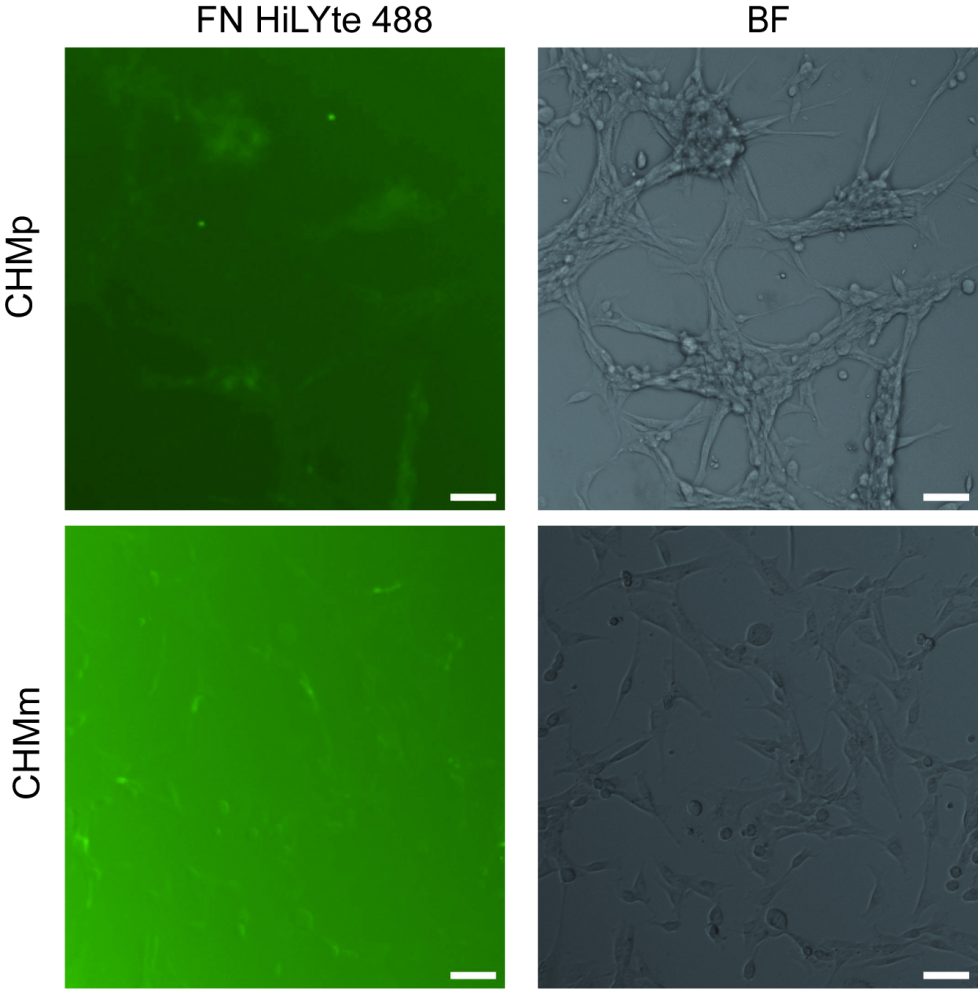
**

**Scale bar: 100 μm**

**Supplementary Table 1** Pool 2 Gene list obtained through screening.

| LMLN |
| --- |
| NRP1 |
| NPM1 |
| RPL3 |
| ANXA1 |
| SH3KBP1 |
| YWHAZ |
| ZFYVE21 |
| DPP4 |
| APBB1IP |
| FLRT2 |
| CTNNA1 |
| ADAM9 |
| PAK2 |
| YWHAG |
| VCL |
| LIMS2 |
| EPHA2 |
| SNTB2 |

**Supplementary Table 2. Specific primers for PCR amplification.**

| **Gene** | **Forward ( 5' -> 3' )** | **Reverse ( 5' -> 3' )** |
| --- | --- | --- |
| RPL13A | GCCGGAAGGTTGTAGTCGT | GGAGGAAGGCCAGGTAATTC |
| CD86 | GGCTTGCCTTTTTCTCGTCC | GCACACCAAGCCACCATTTT |
| CD206 | TCCCAGTGCAGTCCAAACAG | ATTCGCTCTTCGGGTCACAG |
| Arg-1 | ATCTCTGGGGGAGACACCA | AGCAAGCCAGGGTTATTGCT |
| CD44 | GGATGTGCTATGGACGGCTT | CAGGTGGTTGTTGCCTGAGA |
| ADAM9 | TCGAACCTCTGCAAAACAGC | TGTCCTTGTTGGAAACCCCA |
| NANOG | CAGCTTGCCACCACGGAATA | TGGGACACTATCGAGGCAGA |
| CD44 | GGATGTGCTATGGACGGCTT | CAGGTGGTTGTTGCCTGAGA |

**Supplementary Table 2****. Specific electroporation condition forcanine macrophage cell-lines.**

**DH82**

| **Poring Pulse** | **Voltage (V)** | **Pulse Length (msec)** | **Pulse Interval**  **(msec)** | **Number of Pulses** | **Decay Rate**  **(%)** |
| --- | --- | --- | --- | --- | --- |
|  | 125.0 | 5.0 | 50.0 | 2 | 10 |
| **Transfer Pulse** | **Voltage (V)** | **Pulse Length (msec)** | **Pulse Interval**  **(msec)** | **Number of Pulses** | **Decay Rate**  **(%)** |
|  | 20.0 | 50.0 | 50.0 | 5 | 40 |
